# Supplementary material for: Heterologous Expression of the Grapevine JAZ7 Gene in Arabidopsis Confers Enhanced Resistance to Powdery Mildew but Not to Botrytis cinerea
Source: Int J Mol Sci. 2018 Dec 5;19(12):3889. doi: 10.3390/ijms19123889 (PMC6321488; doi:10.3390/ijms19123889)
Supplement: Supplementary file 1 [file ijms-19-03889-s001.pdf]

# Supplementary Information

**Supplementary Data S1:** Analysis of *JAZ7* sequence homology:

```
VqJAZ7    ATGGAGTTTACCCCCAATCTAAGAAAGCAAAACAACTTCCCCTCTGCCCTTCAAGAATCAATCAAGATGGAGAGTAACAAGCCCATGAAT     90
VvJAZ7    ATGGAGTTTACCCCCAATCTAAGAAAGCAAAACAACTTCCCCTCTGCCCTTCAAGAATCAATCAAGATGGAGAGTAACAAGCCCATGAAT     90
Consensusatggagtttacccccaatctaagaaagcaaaacaacttcccctctgcccttcaagaatcaatcaagatggagagtaacaagcccatgaat

VqJAZ7    CTGGAACTTCCACTGTTTCCCTCCACTGCT......TCTTCAATTCCCACTATGAGAGGAGGAGGAGGA.........AGCCCCCAAGAG    165
VvJAZ7    CTGGAACTTCCACTGTTTCCCTCCACTGCTCACACTTCTTCAATTCCCACTATGAGAGGAGGAGGAGGAGGAGGAGGAAGCCCCCAAGAG    180
Consensusctggaacttccactgtttccctccactgctcacacttcttcaattcccactatgagaggaggaggaggaggaggaggaagcccccaagag

VqJAZ7    CAACAGCAACGGCAGCAGCTTACCATCTTCTACAACGGAAGGATTTGCGTTTCCGATGTTACAGAGCTTCGGGCTAGAGCAATTATACTG    255
VvJAZ7    CAACAGCAACGGCAGCAGCTTACCATCTTCTACAACGGAAGGATTTGCGTTTCTGATGTTACAGAGCTTCGGGCTAGAGCAATTATACTG    270
Consensuscaacagcaacggcagcagcttaccatcttctacaacggaaggatttgcgtttccgatgttacagagcttcgggctagagcaattatactg

VqJAZ7    GCTGCAAGTAGAGAAATGGAGGAAAGGAAGAGAGCCCCGCTGTCGCCATCTATGCAATCTCAGCTCTGTGGCCCTTCGGGTGTTTCAATG    345
VvJAZ7    GCTGCAAGTAGAGAAATGGAGGAAAGGAAGAGAGCCCCGCTGTCGCCATCCATGCAATCTCAGCTCTGTGGCCCTTCGGGTGTTTCAATG    360
Consensusgctgcaagtagagaaatggaggaaaggaagagagccccgctgtcgccatccatgcaatctcagctctgtggcccttcgggtgtttcaatg

VqJAZ7    AAGAGATCGCTCCACCGGTTCCTTCAGAAGCGAAAGAATAGGAGGGAAGCTATGTCCCCATACAATCATTAA                      417
VvJAZ7    AAGAGATCGCTCCATCGGTTCCTTCAGAAGCGAAAGAATAGGAGGGAAGCTATGTCCCCATACAATCATTAA                      432
Consensusaagagatcgctccaccggttccttcagaagcgaaagaataggagggaagctatgtccccatacaatcattaa
```

Supplementary Data S1: Multiple alignment of the Open Reading Frame sequences of *JAZ7* genes from *V. quinquangularis* and *V. vinifera*. The *VqJAZ7* cDNA was predicted to be 417 bp in length, which is 15 bp shorter than *VvJAZ*.

**Supplementary Data S2:** Analysis of *JAZ7* protein sequence alignment

```
VqJAZ7    MEFTPNLRKQNNFPSALQESIKMESNKPMNLELPLFPSTA..SSIPTMRGGGG...SPQEQQQRQQLTIFYNGRICVSDVTELRARAIIL     85
VvJAZ7    MEFTPNLRKQNNFPSALQESIKMESNKPMNLELPLFPSTAHTSSIPTMRGGGGGGGSPQEQQQRQQLTIFYNGRICVSDVTELRARAIIL     90
Consensusmeftpnlrkqnnfpsalqesikmesnkpmnlelplfpstahtssiptmrgggggggspqeqqqrqqltifyngricvsdvtelraraiil

VqJAZ7    AASREMEERKRAPLSPSMQSQLCGPSGVSMKRSLHRFLQKRKNRREAMSPYNH                                        138
VvJAZ7    AASREMEERKRAPLSPSMQSQLCGPSGVSMKRSLHRFLQKRKNRREAMSPYNH                                        143
Consensusaasremeerkraplspsmqsqlcgpsgvsmkrslhrflqkrknrreamspynh
```

Supplementary Data S2: Multiple alignment of amino acid sequences of *JAZ7* genes from *V. quinquangularis* and *V. vinifera*.

**Supplementary Data S3:** Analysis of The ORF sequence of *VqJAZ7* gene

```
1      ATGGAGTTTACCCCCAATCTAAGAAAGCAAAACAACTTCCCCTCTGCCCTTCAAGAATCA
1       M  E  F  T  P  N  L  R  K  Q  N  N  F  P  S  A  L  Q  E  S
61     ATCAAGATGGAGAGTAACAAGCCCATGAATCTGGAACTTCCACTGTTTCCCTCCACTGCT
21      I  K  M  E  S  N  K  P  M  N  L  E  L  P  L  F  P  S  T  A
121    TCTTCAATTCCCACTATGAGAGGAGGAGGAGGAAGCCCCCAAGAGCAACAGCAACGGCAG
41      S  S  I  P  T  M  R  G  G  G  G  S  P  Q  E  Q  Q  Q  R  Q
181    CAGCTTACCATCTTCTACAACGGAAGGATTTGCGTTTCCGATGTTACAGAGCTTCGGGCT
61      Q  L  T  I  F  Y  N  G  R  I  C  V  S  D  V  T  E  L  R  A
241    AGAGCAATTATACTGGCTGCAAGTAGAGAAATGGAGGAAAGGAAGAGAGCCCCGCTGTCG
81      R  A  I  I  L  A  A  S  R  E  M  E  E  R  K  R  A  P  L  S
301    CCATCTATGCAATCTCAGCTCTGTGGCCCTTCGGGTGTTTCAATGAAGAGATCGCTCCAC
101     P  S  M  Q  S  Q  L  C  G  P  S  G  V  S  M  K  R  S  L  H
361    CGGTTCCTTCAGAAGCGAAAGAATAGGAGGGAAGCTATGTCCCCATACAATCATTAA
121     R  F  L  Q  K  R  K  N  R  R  E  A  M  S  P  Y  N  H  *
```

Supplementary Data S3: The ORF sequence of *VqJAZ7* gene in *V. quinquangularis* and its coded amino acid sequence:

Black letters for the nucleotide sequence of ORF， blue letters for the deduced amino acid sequence, red letters for the conserved domain of *VqJAZ7*, the underlined letters for the start and stop codons of ORF.

**Supplementary Table S1:** Primers used for disease resistance analysis in *A. thaliana*

| Genes | Gene Locus ID | Forwards primers | Reverse primers |
|---|---|---|---|
| *AtPR1* | AT2G14610 | AACTACGCTGCGAACACGTG | TCACTTTGGCACATCCGAGTC |
| *AtICS1* | AT1G74710 | CTTCCGTGACCTTGATCCTTTCT | CAGCGATCTTGCCATTAGGATC |
| *AtPDF1.2* | AT5G44420 | GAAGCACAGAAGTTGTGCGA | TGTAACAACAACGGGAAAATAAACA |
| *AtLOX3* | AT2G35980 | TCTCCGTACAACAAGCGTTGG | GCGTCCGTCTAGCGCATTAAT |
| *AtActin* | AT2G37620 | AGTGTCTGGATCGGTGGTTC | CCCCAGCTTTTTAAGCCTTT |

**Supplementary Figure S1:** Characterization of the *VqJAZ7* over-expression in Arabidopsis

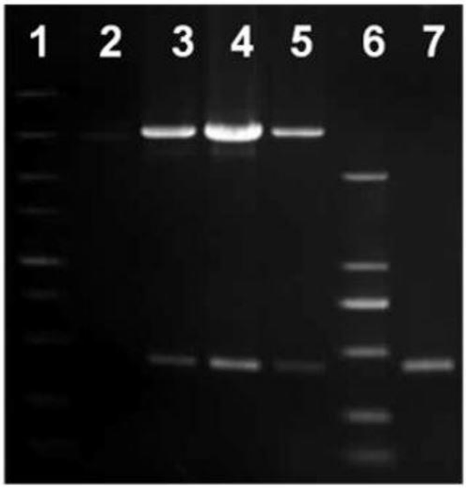


Supplementary Figure S1: Characterization of the *VqJAZ7* over-expression in *A. thaliana* lines:

Double digestion of recombinant pGEM-T/*VqJAZ7* vector, Lane 1: DNA Marker DL5000, Lane 2: recombinant pGEM-T/*VqJAZ7* vector, Lane 3-5: recombinant vector pGEM-T/*VqJAZ7* was digested by XbaI and KpnI, Lane 6: DNA Marker DL2000, Lane 7: ORF of *VqJAZ7* gene.
